# Annealing Effects on Structure and Optical Properties of Diamond-Like Carbon Films Containing Silver

**DOI:** 10.1186/s11671-016-1362-4

**Published:** 2016-03-15

**Authors:** Šarūnas Meškinis, Arvydas Čiegis, Andrius Vasiliauskas, Kęstutis Šlapikas, Rimantas Gudaitis, Iryna Yaremchuk, Volodymyr Fitio, Yaroslav Bobitski, Sigitas Tamulevičius

**Affiliations:** Institute of Materials Science of Kaunas University of Technology, Barsausko 59, LT-51423 Kaunas, Lithuania; Department of Photonics, Lviv Polytechnic National University, S. Bandera Str. 12, Lviv, 79013 Ukraine; Faculty of Mathematics and Natural Sciences, University of Rzeszow, Pigonia Str.1, 35959 Rzeszow, Poland

**Keywords:** Diamond-like carbon, Silver nanoparticles, Nanocomposite, Surface plasmon resonance, Annealing

## Abstract

In the present study, diamond-like carbon films with embedded Ag nanoparticles (DLC:Ag) were deposited by reactive magnetron sputtering. Structure of the films was investigated by Raman scattering spectroscopy. Atomic force microscopy was used to define thickness of DLC:Ag films as well as to study the surface morphology and size distribution of Ag nanoparticles. Optical absorbance and reflectance spectra of the films were studied in the 180–1100-nm range. Air annealing effects on structure and optical properties of the DLC:Ag were investigated. Annealing temperatures were varied in the 180–400 °C range. Changes of size and shape of the Ag nanoclusters took place due to agglomeration. It was found that air annealing of DLC:Ag films can result in graphitization following destruction of the DLC matrix. Additional activation of surface-enhanced Raman scattering (SERS) effect in DLC:Ag films can be achieved by properly selecting annealing conditions. Annealing resulted in blueshift as well as significant narrowing of the plasmonic absorbance and reflectance peaks. Moreover, quadrupole surface plasmon resonance peaks appeared. Modeling of absorption spectra of the nanoclusters depending on the shape and surrounding media has been carried out.

## Background

Surface plasmons are collective oscillations of the free electrons localized at surfaces of metal structures [1]. At a certain excitation frequency, these oscillations will be in resonance with the incident light, resulting in significantly increased intensity of oscillation of the surface electrons [2]. This effect is called localized surface plasmon resonance (LSPR) [2]. Surface plasmon resonance effect results in resonant optical absorbance, transmittance, scattering, and/or reflectance spectra of the plasmonic materials [1, 2]. The most often used plasmonic materials are the group IB metal (gold, silver, copper) nanoparticles. Position of the nanoparticle surface plasmon resonance peak can be controlled by setting the appropriate nanoparticle shape, size, and interparticle distance [1]. Among all of the group IB metals, silver has some advantages over Au and Cu as a plasmonic material [3, 4]. Particularly, the stronger surface plasmon resonance effect should be mentioned [3, 4].

An interesting class of plasmonic materials is plasmonic nanocomposites [5]. These nanomaterials consist of a dielectric matrix with the embedded metal nanoparticles [5]. Using such nanocomposites, Ag nanoparticle oxidation problems can be controlled or even avoided [6–8]. It provides additional possibility to control the optical properties of the plasmonic material by using a dielectric matrix material of the appropriate dielectric permittivity [5]. The prospective dielectric matrix material is diamond-like carbon (DLC). DLC is an amorphous allotrope of carbon consisting of the sp^3^-bonded and sp^2^-bonded carbon atoms and containing 0–40 at.% of hydrogen [9, 10]. Hardness of these films is as high as up to 80 % of the diamond hardness [9, 10]. Wear and corrosion resistance as well as biocompatibility can be mentioned, too [9, 10]. Optical and electrical properties of DLC films can be changed in a broad range [9, 10]. DLC films containing silver (DLC:Ag) are growing in the form of the nanocomposites with embedded Ag nanoparticles when the silver atomic concentration in the film is more than 1–2 at.% [11–13]. Surface plasmon resonance effect was reported for the DLC:Ag films [14].

Optical properties of the plasmonic nanocomposites and other plasmonic nanomaterials can be additionally changed by using annealing [15–20]. In such a way, increased intensity of the surface plasmon resonance absorption [15, 18, 19] and reflectance [20] peaks as well as control of the plasmonic peak position [17, 19] can be achieved. Using such approach, new interesting nanostructures such as ZnO nanorods with embedded Au nanoparticles can be fabricated [15]. However, annealing can result in detrimental effects on optical properties of the plasmonic nanocomposites, too [16, 17]. Thus, in every case, the annealing process should be optimized, taking into account properties of the dielectric matrix. However, there are no studies on the influence of annealing on optical properties of the plasmonic DLC nanocomposites with embedded group IB metal nanoparticles. Therefore, in the present research, annealing effects on the structure and optical properties of DLC:Ag were studied. Modeling and experimental research were combined to elucidate processes taking place during the annealing.

## Methods

In the present study, DLC:Ag were deposited by using reactive direct current unbalanced magnetron sputtering of the silver target. The diameter of the magnetron was 3 in. In most cases, silica substrates were used. Samples for the study of the Raman scattering spectra were grown on monocrystalline silicon substrates. Mixture of the hydrocarbons (acetylene) and argon gas was used in the reactive magnetron sputtering system. Ar gas flux was 80 sccm and C_2_H_2_ gas flux was 7.8 sccm. In all the experiments, the substrate-target gap was set at 10 cm, magnetron target current was 0.1 A, base pressure was 5 × 10^−4^ Pa, and work pressure was (4 ± 1) × 10^−1^ Pa. The thickness of the deposited films was about 50 nm. No additional bias during deposition was used, and the substrates were grounded. For more information on deposition conditions as well as chemical composition, structure, and optical properties of DLC:Ag films, please see [14].

After the deposition, DLC:Ag films were annealed in air at 140, 200, 300, and 400 °C temperatures. In all cases, annealing time was 3 min. Annealing was conducted using a muffle furnace. It was preheated to the necessary temperature before the DLC:Ag films were placed inside. Subsequent temperature stabilization took place in a 30–60-s time interval. Afterwards, annealing was carried out for 3 min. After the annealing, the samples were immediately taken from the furnace and were cooled to room temperature (~20 °C) for 5–10 min. After that, the films were investigated.

The morphology of the thin film surface was analyzed by the atomic force microscope (AFM) NanoWizard® 3 (JPK, Germany) working in AC mode. Silicon probes with a reflective backside Al coating (ACTA-10, AppNano, USA) with a resonance frequency of 200–400 kHz and force constant of 13–77 N/m were used. Nominal tip radius was less than 10 nm. The scanning rate of 0.8 Hz was selected.

Thickness of the thin films was evaluated by fabrication of the DLC:Ag film-based diffraction grating and measurement of the grating step height by using AFM.

Raman scattering measurements were performed using the Raman microscope inVia (Renishaw) with a 532-nm excitation. Integration time was 100 s, power was 0.3 mW, and grating groove density was 2400 grooves/mm.

The Martens hardness of the nanocomposite films was measured by using the micro-indenter Fischerscope HM2000 (Helmut Fischer GmbH, Germany). A Berkovich-type diamond indentor was used. In all cases, a 0.4-mN load was applied. Ten repeat indentations were made for each sample, and hardness value is given as an average of all the measurements.

Optical properties of the DLC:Ag in the 180–1100-nm range were investigated by using the fiber optic spectrometer AvaSpec-2048 (Avantes). The spectrometer is based on the AvaBench-75 symmetrical Czerny-Turner design and is equipped with a 2048-pixel CCD detector array (resolution 1.4 nm).

The electrodynamic dipole approaches were used for simulation of the optical properties of plasmonic nanocomposites.

## Results and Discussion

**Fig. 1:**
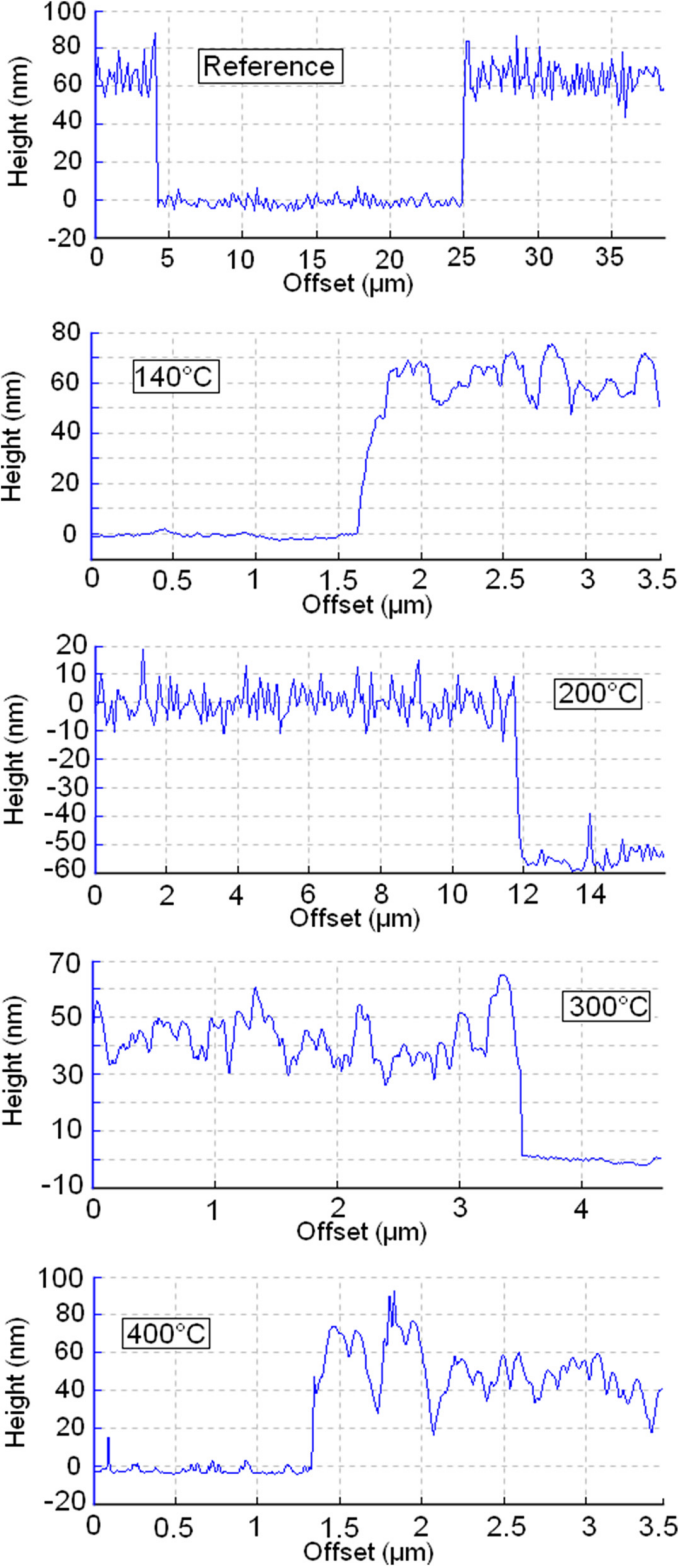
Annealing effects on the DLC:Ag grating step height

**Fig. 2:**
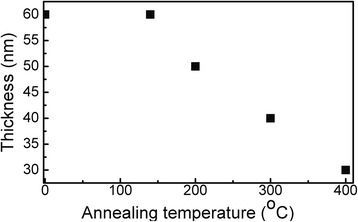
Annealing effects on thickness of DLC:Ag films. *0 °C* point refers to the initial thickness of the film (before annealing)

In the present research, as a first step, influence of the annealing on the thickness of DLC:Ag films was studied. It can be seen in Figs. 1 and 2 that air annealing at 140 °C temperature does not change the thickness of the DLC:Ag thin film. However, further annealing at higher temperatures resulted in decreased thickness of the DLC:Ag film.

**Fig. 3:**
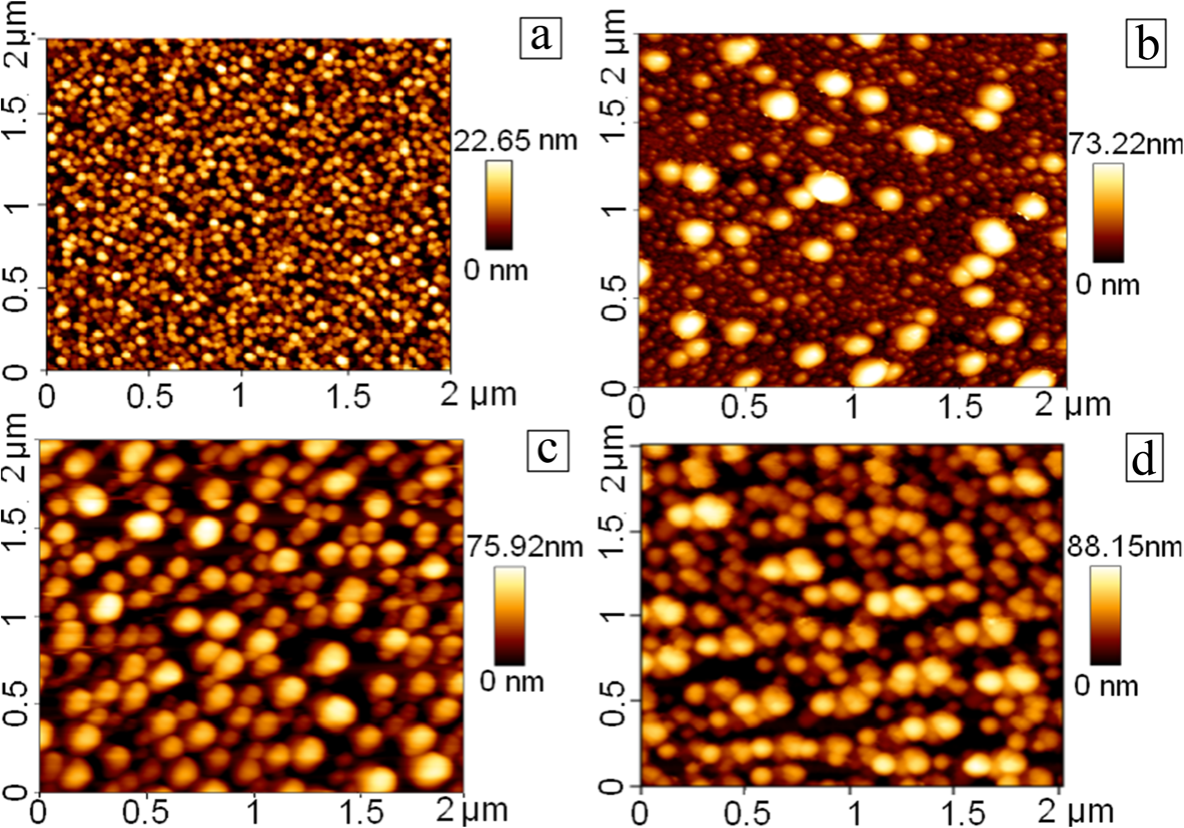
AFM images of DLC:Ag films: reference (**a**), after annealing at 200 °C (**b**), after annealing at 300 °C (**c**), and after annealing at 400 °C (**d**)

AFM images of the DLC:Ag films are presented in Fig. 3. In all cases, circularly shaped nanoclusters can be seen. In our previous study, we have demonstrated that the observed circular nanofeatures are Ag nanoclusters and that atomic force microscopy can be used to measure the size of the silver nanoclusters embedded into the DLC:Ag film [21]. One can see that circular nanoclusters with less than 50 nm in diameter prevail in the case of the reference sample. Larger Ag nanocluster (>100 nm) diameters along with smaller ones appear as a result of the annealing at 200 °C temperature. Some of the larger nanoclusters change shape and become prolonged. Annealing at 300 °C temperature results in disappearance of the small nanoclusters. The prolonged nanoclusters with a diameter higher than 100 nm prevail. Thus, coalescence of the Ag nanoparticles takes place as a result of the annealing.

**Fig. 4:**
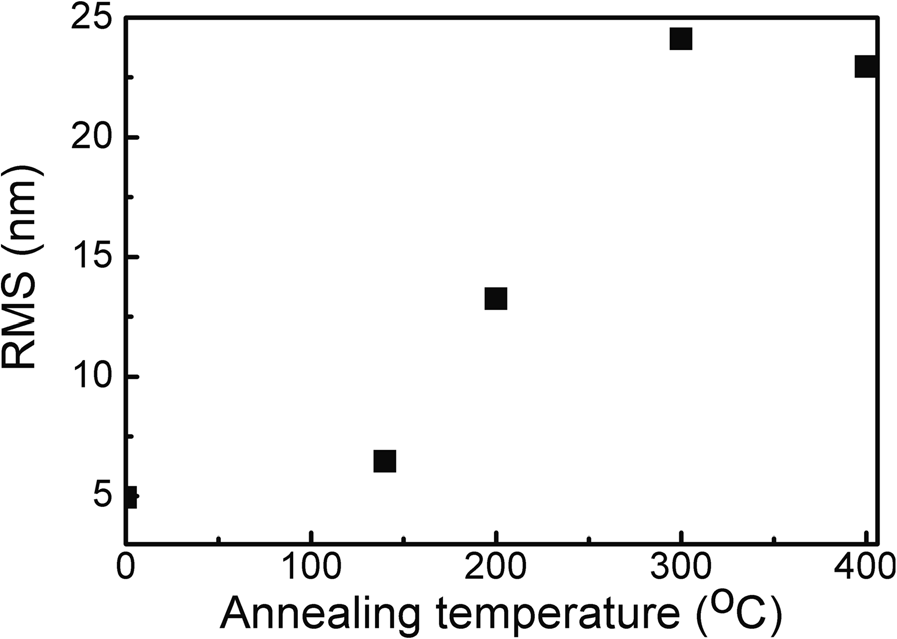
Annealing effects on the surface roughness of DLC:Ag films

Annealing at 200 °C and higher temperatures results in significant increase of the sample roughness as well (e.g., from 5 to 22 nm for the reference sample and sample annealed at 400 °C, respectively (Fig. 4)).

**Fig. 5:**
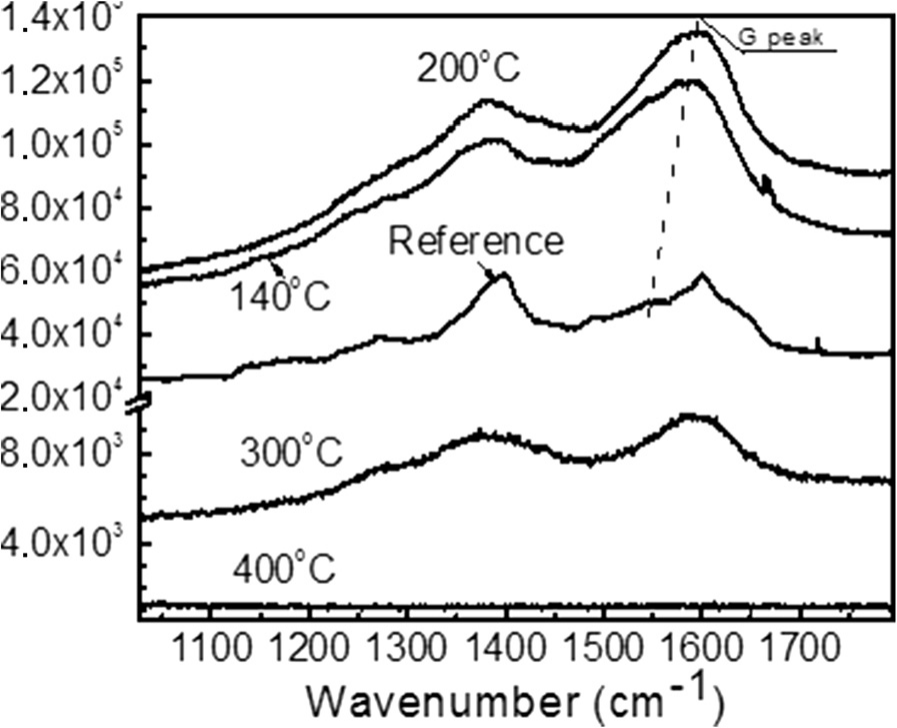
Raman scattering spectra of DLC:Ag film: reference and after the annealing at different temperatures

Raman scattering spectra of the DLC:Ag film annealed at different temperatures are presented in Fig. 5. In the case of the reference sample (before annealing), surface-enhanced Raman scattering (SERS) effect can be clearly seen. A lot of the peaks in addition to the D and G peaks typical for DLC can be seen. Annealing at 140 and 200 °C temperatures resulted in increased intensity of the Raman scattering spectra and G peak particularly. However, additional peaks disappeared or became less intensive. Increase of the annealing temperature to 300 °C resulted in significant decrease of the intensity of Raman scattering peaks. In comparison with the reference sample, intensity decreased approximately five times. Changes of intensity of the Raman scattering can be explained by the changes of Ag nanoparticle size as well as interparticle distance. For example, in [22], it was shown that SERS signal of adenine molecules deposited on an ordered Ag nanoparticle array increases with the increased particle size and decreased with the interparticle distance. In our case, SERS intensity increases with annealing at 140 and 200 °C. However, further increase of the annealing temperature results in the decreased intensity despite increased size of Ag nanoparticles. It can be seen in Fig. 3 that after the annealing at 300 °C temperature, interparticle distance increases along with the Ag nanocluster size. Thus, increased interparticle distance results in the decreased intensity of SERS signal.

Further increase of the annealing temperature up to 400 °C resulted in disappearance of the Raman scattering peaks in the investigated spectral range. It seems that at this temperature, a structure typical of the DLC is destructed or the DLC matrix is burned out (vaporized). This permission is in good accordance with the decreased thickness of the film as a result of the annealing (Figs. 1 and 2) as well as increased surface roughness (Fig. 4). In addition, shift of the G peak position to the higher wave numbers should be mentioned. According to [9], it means decreased sp^3^/sp^2^ carbon bond ratio (graphitization of the amorphous carbon matrix). This fact is in good accordance with the data reported by other studies on annealing of undoped DLC films [23, 24]. In [23], annealing of undoped hydrogenated DLC films in oxygen-containing ambient resulted in mass loss, decrease of thickness, and sp^3^/sp^2^ carbon bond ratio decrease of the hydrogenated DLC film.

The Martens hardness of the DLC:Ag films before the annealing was 8.407 ± 1.495 N/mm^2^. It should be mentioned that similar hardness was reported in [25] for DLC:Ag nanocomposite films containing ~13 at.% Ag. Film annealing resulted in the decreased hardness, and for example, after annealing at 140 °C temperature, it was decreased twice and was equal to 4.115 ± 0.689 N/mm^2^. This decrease is in good accordance with the decrease of the sp^3^/sp^2^ carbon bond ratio mentioned above.

**Fig. 6:**
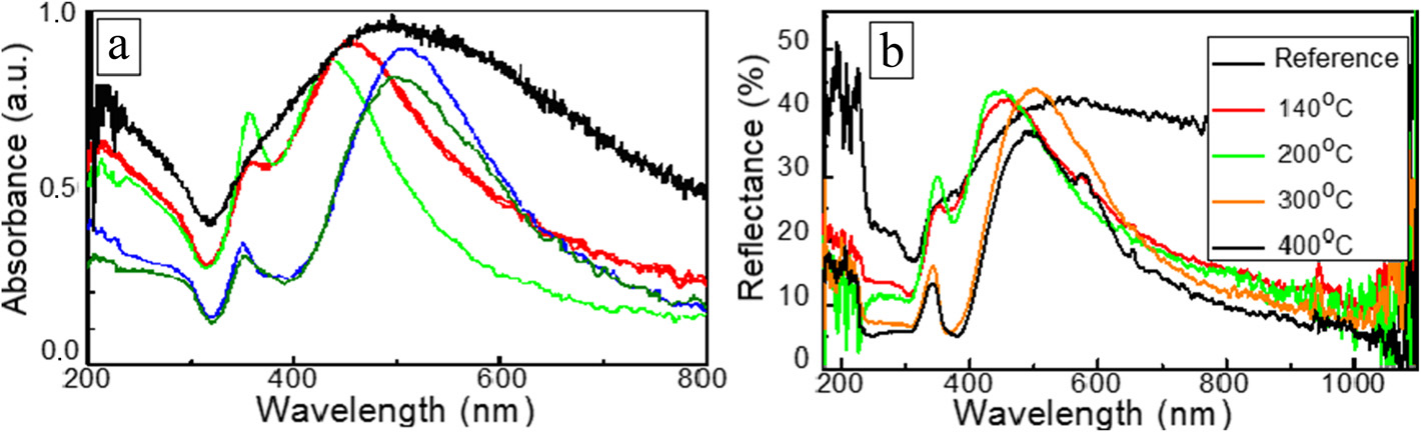
Influence of the annealing on optical absorbance (**a**) and reflectance (**b**) spectra of the DLC:Ag films

Optical absorbance and reflectance spectra of the DLC:Ag film annealed at different temperatures are presented in Fig. 6. One can see that after annealing plasmon peaks shifted and additional peaks can be clearly distinguished (Fig. 3). It should be noted as well that annealing at 140 and 200 °C temperatures resulted in blueshift of the surface plasmon resonance peak and a substantial decrease of their width. Moreover, the second absorption peak at a <400-nm wavelength appeared. Increase of the annealing temperature up to 300 and 400 °C resulted in redshift of the main surface plasmon resonance peak. Similar behavior of the plasmonic peak was found in the reflectance spectra. All these regularities observed can be explained by the changes of the average size of Ag nanoclusters. In all cases, annealing resulted in the decreased width of the main plasmonic absorbance peak.

As it was indicated, the larger Ag nanoclusters along with the smaller ones appear as a result of the annealing (Fig. 3). The shape of these nanoclusters plays a crucial role in the absorption spectra, since we assign the peak in the absorption spectra to the plasmon excited in the clusters of certain size. It is known that for a spheroid-shaped metallic nanoparticle, the plasmon band splits into two bands corresponding to the oscillation of the free electrons along (dipole plasmon) and perpendicular (quadrupole plasmon) to the long axis of the spheroid [26, 27]. For small nanoparticles, only dipole resonance modes can be excited, whereas for larger nanoparticles, quadrupole resonance modes can also be excited because of the onset of the electromagnetic retardation effect [28]. Moreover, the similar dipole and quadrupole plasmon resonant wavelengths already have been observed experimentally for Ag-SiO_2_ nanostructures in [29].

Usually, the decreased nanoparticle size results in blueshift of the plasmonic peak [1, 14, 21]. On the other hand, in our case when we are dealing with the nanocomposite, the decreased thickness of the DLC matrix should be taken into account, too. It should result in an effect similar to that of the decreased refractive index of the media surrounding Ag nanoparticles. For example, the decreased refractive index in such a case results in blueshift of the plasmonic peak [5], too. So blueshift of the plasmonic peak observed is in good accordance with this assumption [1, 24].

The simulation was done to clarify observed changes of the optical properties of the DLC:Ag films. Let us assume that small spherical nanoparticles stick together and form large nanoclusters having a shape of spheroids. In such a way, it is easy to explain the appearance of two peaks of absorption. The two resonances corresponding to the oscillations of electrons across and along the axis of symmetry of the particles appear for the spheroidal nanoparticles [26]. Of course, the shape of these particles is not perfectly spheroidal and their dimensions differ slightly from each other. However, such spheroidal approach seems quite appropriate, because it allows a more or less accurate description of the properties of these particles. The absorption of the nanoclusters can be calculated using electrodynamic dipole approaches as follows [27]:

1 $$ {C}_{\mathrm{abs}}=\frac{12\pi k{\varepsilon}_h\mathrm{I}\mathrm{m}\left({\varepsilon}_i\right)}{R^3{\left|{\varepsilon}_i-{\varepsilon}_h\right|}^2}{\left|{\alpha}_{\perp, \mathrm{I}\mathrm{I}}\right|}^2, $$

where *ε*_*i*_ and *ε*_*h*_ are dielectric permittivity of nanoparticles and the surrounding medium, respectively.

Tensor of polarizability nonspherical nanoparticles is given as follows [28]:

2 $$ {\alpha}_{\perp, \mathrm{I}\mathrm{I}}=\frac{2{\alpha}_{\perp }+{\alpha}_{\mathrm{II}}}{3}, $$

where components *α*_⊥_ and *α*_II_ are given as follows:

3 $$ {\alpha}_{\perp }=\frac{\varepsilon_i/{\varepsilon}_h-1}{\left({\varepsilon}_i/{\varepsilon}_h-1\right)\left(\frac{1}{3}\mp \frac{1}{15}{e}^2\right)+1}\left(\frac{V}{4\pi}\right), $$

4 $$ {\alpha}_{\mathrm{II}}=\frac{\varepsilon_i/{\varepsilon}_h-1}{\left({\varepsilon}_i/{\varepsilon}_h-1\right)\left(\frac{1}{3}\pm \frac{2}{15}{e}^2\right)+1}\left(\frac{V}{4\pi}\right), $$

where *e* is nanoparticle eccentricity (two signs correspond to prolate or oblate shape).

**Fig. 7:**
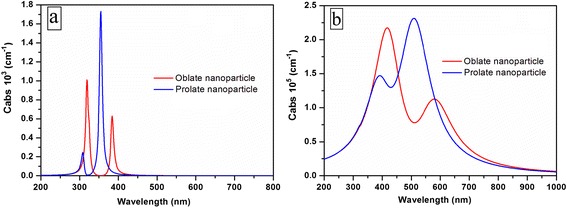
Resonance responses of the silver nanoparticles of elliptical shape (prolate or oblate) in air (**a**) and in DLC (**b**)

The calculated absorption cross sections of spheroidal particles (prolate and oblate) in air and DLC film are presented in Fig. 7a, b, respectively. The eccentricity in these calculations was arbitrarily selected to be 0.9. In the calculations, refractive index of the substrate (fused silica) was calculated using dispersion equations [29]. The dielectric constant of silver was used from [30]. We have used optical constants of the DLC film from [22] that were deposited at similar conditions.

According to the calculations, nanoparticles placed in air have two narrow absorption peaks near 300 and 360 nm for the prolate shape and near 320 and 390 nm for the oblate. Two peaks in the absorption spectra of nanoclusters placed in the DLC are shifted to the long-wavelength region—380 and 520 nm for the prolate and 420 and 590 nm for the oblate. Moreover, the peaks are broadened.

**Fig. 8:**
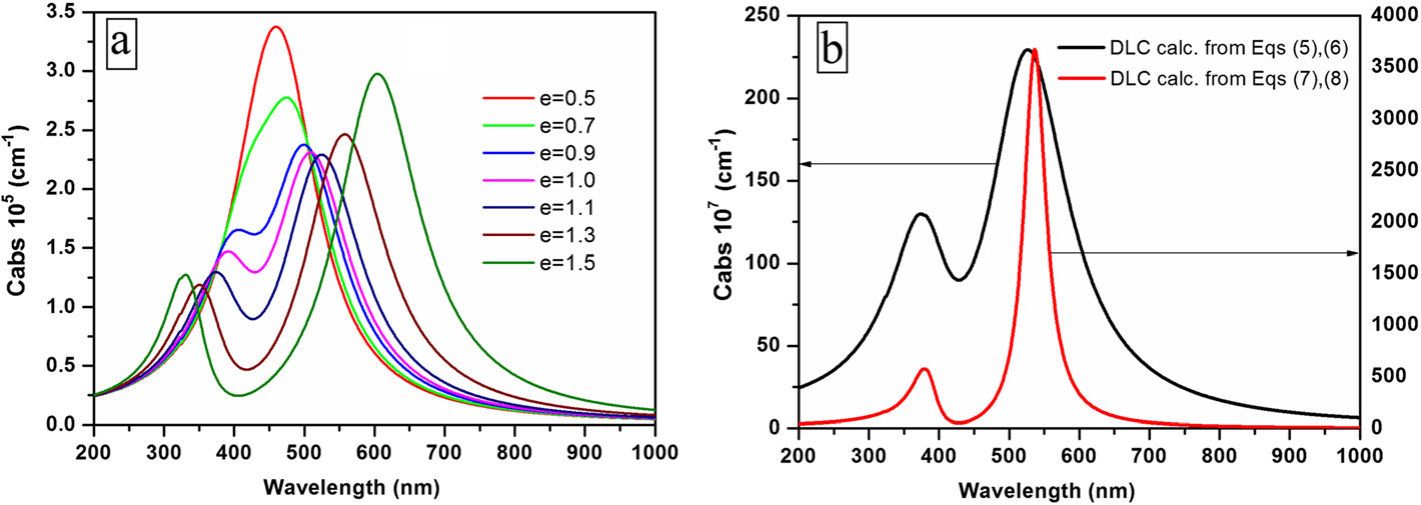
Resonance responses of the silver nanocluster with different eccentricities (**a**) and different refractive indices (**b**) of DLC

Comparison of the experimental results (see Fig. 6) with the obtained ones allows us to suppose that prolate nanoclusters dominate in our samples. Thus, we will use this shape in further calculations. The nanoclusters in DLC films after annealing do not have the ideal spheroid shape. Thus, it is necessary to study the influence of the eccentricity on the absorption cross-section spectrum at the constant refractive index of the DLC. Fig. 8a demonstrates that an additional quadrupole peak appears for the nanoclusters with eccentricity 0.9 in the surrounding media (DLC) with optical constant taken from [31]. We have used values of the refractive index and extinction coefficient of DLC that were taken from the experimental dispersion curves and extrapolated by fifth-order polynomial to fit the measured data as follows:

5 $$ \begin{array}{l}n=1.30568+0.00426\lambda -1.04027\times {10}^{-5}{\lambda}^2+\\ {}\kern1.5em +1.12199\times {10}^{-8}{\lambda}^3-5.61906\times {10}^{-12}{\lambda}^4+1.06725\times {10}^{-15}{\lambda}^5\end{array} $$

6 $$ \begin{array}{l}k=0.61709-0.00168\lambda +8.79418\times {10}^{-7}{\lambda}^2+\\ {}\kern1.5em +1.1057\times {10}^{-9}{\lambda}^3-1.2658\times {10}^{-12}{\lambda}^4+3.41168\times {10}^{-16}{\lambda}^5\end{array} $$

Increasing of the eccentricity results in the increased intensity of the peaks and increased distance between them. To study the influence of the surrounding media, in the present calculations, we have used the two different refractive indices of DLC film—in addition to the set optical constant calculated by Eqs. (5) and (6), we have used as well the optical constant (from [22]) that was extrapolated by fifth-order polynomial as follows:

7 $$ \begin{array}{l}n=0.99176+0.00599\lambda -1.36751\times {10}^{-5}{\lambda}^2+\\ {}\kern1.5em +1.41206\times {10}^{-8}{\lambda}^3-6.85141\times {10}^{-12}{\lambda}^4+1.26971\times {10}^{-15}{\lambda}^5\end{array} $$

8 $$ \begin{array}{l}k=0.21748-0.00219\lambda +9.57262\times {10}^{-6}{\lambda}^2+\\ {}\kern1.5em +1.33692\times {10}^{-8}{\lambda}^3-7.84873\times {10}^{-12}{\lambda}^4+1.66521\times {10}^{-15}{\lambda}^5\end{array} $$

One can see that the absorption peaks are sensitive to the dielectric constant of DLC—peaks (Fig. 8b) are different in value of absorption, peak positions, and bandwidth.

**Fig. 9:**
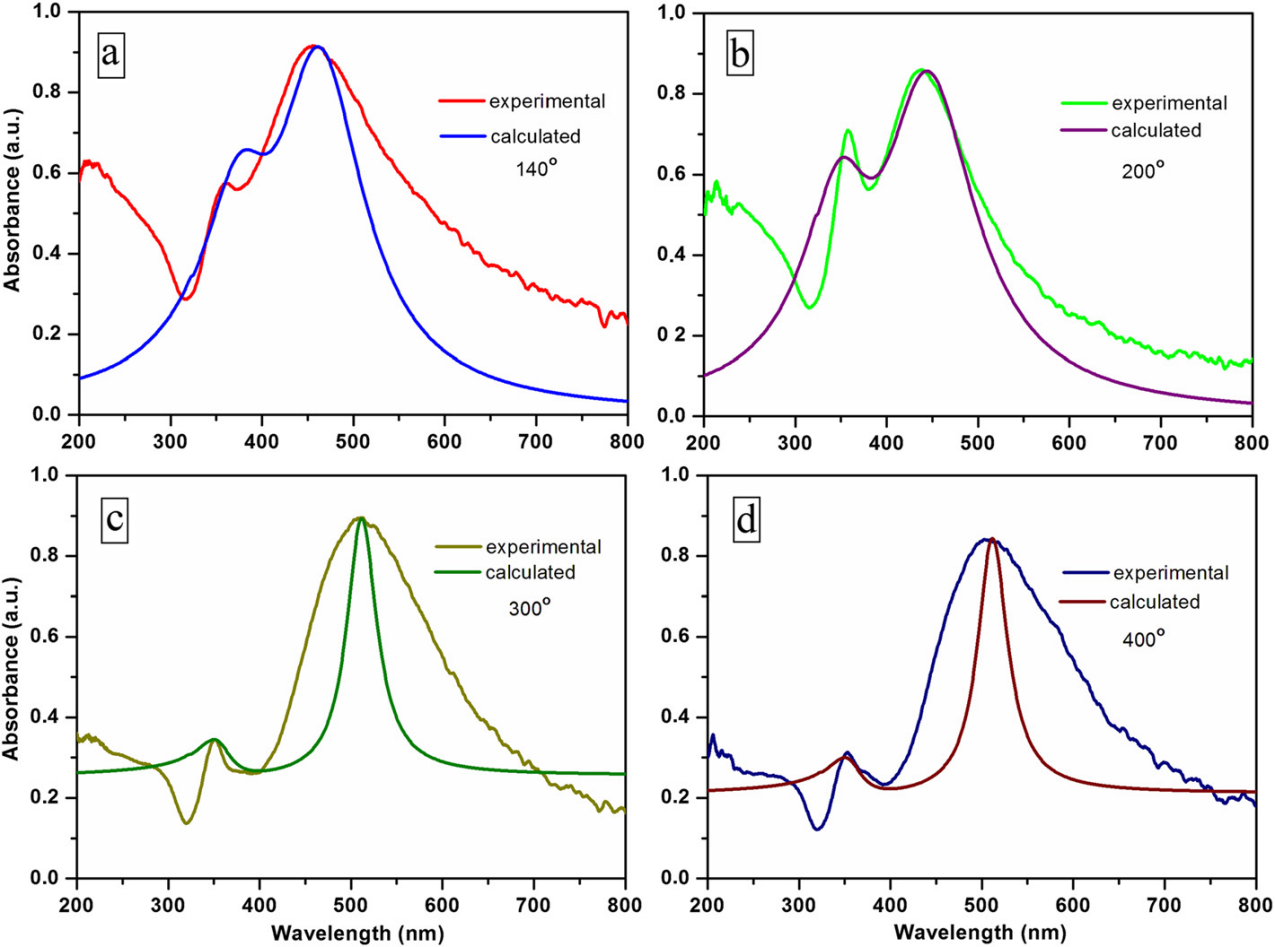
Experimental and calculated optical absorbance of DLC film doped by silver nanoparticles after annealing at 140 (**a**), 200 (**b**), 300 (**c**), and 400 °C (**d**)

To confirm the assumption that the formed after-annealing nanoclusters having nonspherical shape play the main role in the absorption, we calculated the absorption spectra of the nanocomposite films after annealing at temperatures of 140, 200, 300, and 400 °C, and the results of the calculations are shown in Fig. 9.

Having the size of nanoclusters from AFM (Fig. 3) and experimental value of DLC refractive index (measured at 632 nm), we fitted eccentricity of the formed nanoclusters after annealing at different temperatures. In this way, we have obtained optimal fitting of the experimental and calculated data for annealing: at 140 °C, *e* = 0.89 (Fig. 9a); at 200 °C, *e* = 1.0 (Fig. 9b); and at 300 and 400 °C, *e* = 1.2 (Fig. 9c, d, respectively). The results show that with the increase in annealing temperature, the nanoclusters became more and more asymmetrical. A large discrepancy between the bandwidth of the calculated and experimental curves presented in Fig. 9c, d can be explained by the dominant role of large nanoclusters in the absorption spectrum. According to our calculations, the nanoclusters obtained after annealing by 300 and 400 °C have the same asymmetry; however, the refractive index of DLC is slightly changed, resulting in two well-expressed absorption peaks.

In summary, change of the absorption spectra of the DLC:Ag film as a result of the annealing is caused by changes of the size of nanoparticles due to their coalescence and change of the relative permittivity of the surrounding host media.

It should be mentioned that annealing-induced appearance of the second plasmonic peak can be beneficial in different applications. Particularly in SERS applications, it can result in simultaneous enhancement at both the excitation and the specific Raman-Stokes lines [32]. Double plasmon resonance can be used as well for fabrication of the double wavelength chemical sensors [33]. Narrowing of the plasmonic peak width results in the increased resolution of the sensor [34]. Thus, by setting appropriate annealing conditions, modification of the optical properties advantageous for the sensor applications takes place.

## Conclusions

Thermal air annealing in the 140–400 °C temperature range resulted in the decreased thickness of DLC:Ag films and increased surface roughness. Decrease of sp^3^/sp^2^ carbon bond ratio (graphitization of the amorphous carbon matrix) took place. Air annealing at 400 °C resulted in destruction of the DLC matrix. The size of Ag nanoparticles embedded into the DLC:Ag matrix increased with the annealing temperature. Their shape has been changed from spherical to the prolated. This effect was explained by agglomeration of the Ag nanoparticles.

Annealing at 140 and 200 °C temperatures resulted in increased intensity of the Raman scattering spectra and G peak particularly. Further increase of the annealing temperature resulted in significant decrease of the intensity of carbon-related Raman scattering peaks and even their disappearance.

Annealing at 140 and 200 °C temperatures resulted in blueshift of the surface plasmon resonance peak and substantially decreased their width. The second absorption peak at a <400-nm wavelength appeared. Increase of the annealing temperature to 300 and 400 °C resulted in redshift of the main surface plasmon resonance peak. A similar behavior was observed in the case of the reflectance spectra. Shift of the absorption spectra and appearance of the additional quadrupole peaks were studied by using a combination of the modeling and experimental studies. The observed regularities were explained by the increased size of the nanoparticles, formation of the prolated nanoparticles instead of spheroidal ones, and change of the relative permittivity of the surrounding media.

## References

[1] Hayashi S, Okamoto T (2012). Plasmonics: visit the past to know the future. J Phys D Appl Phys.

[2] Rycenga M, Cobley CM, Zeng J, Li W, Moran CH, Zhang Q, Qin D, Xia Y (2011). Controlling the synthesis and assembly of silver nanostructures for plasmonic applications. Chem Rev.

[3] Pena-Rodríguez O, Pal U (2011). Enhanced plasmonic behavior of bimetallic (Ag–Au) multilayered spheres. Nanoscale Res Lett.

[4] Kyeong-Seok Lee M, El-Sayed A (2006). Gold and silver nanoparticles in sensing and imaging: sensitivity of plasmon response to size, shape, and metal composition. J PhysChem B.

[5] Hedayati MK, Faupel F, Elbahri M (2014). Review of plasmonic nanocomposite metamaterial absorber. Materials.

[6] Formo EV, Mahurin SM, Sheng D (2010). Robust SERS substrates generated by coupling a bottom-up approach and atomic layer deposition. ACS Applied Material Interfaces.

[7] Xiaoyu Z, Jing Z, Whitney AV, Elam JW, Van Duyne RP (2006). Ultrastable substrates for surface-enhanced Raman spectroscopy: Al2O3 overlayers fabricated by atomic layer deposition yield improved anthrax biomarker detection. J Am Chem Soc.

[8] Li JF, Huang YF, Ding Y, Yang ZL, Li SB, Zhou XS, Fan FR, Zhang W, Zhou ZY, Wu DY, Ren B, Wang ZL, Tian ZQ (2010). Shell-isolated nanoparticle-enhanced Raman spectroscopy. Nature.

[9] Robertson J (2002). Diamond like amorphous carbon. Mater Sci Eng R.

[10] Robertson J (2008). Comparison of diamond-like carbon to diamond for applications. Phys Status Solidi A.

[11] Choi HW, Choi J-H, Lee K-R, Ahn J-P, Oh KH (2007). Structure and mechanical properties of Ag-incorporated DLC films prepared by a hybrid ion beam deposition system. Thin Solid Films.

[12] Baba K, Hatada R, Flege S, Ensinger W. (2011) Preparation and properties of Ag-containing diamond-like carbon films by magnetron plasma source ion implantation. Adv in Mat Sci Eng, 2012: 536853-536858

[13] Choi HW, Dauskardt RH, Lee S-C, Lee K-R, Oh KH (2008). Characteristic of silver doped DLC films on surface properties and protein adsorption. Diamond Related Mater.

[14] Meškinis Š, Čiegis A, Čiegis A, Vasiliauskas A, Tamulevičienė A, Šlapikas K, Juškėnas R, Niaura G (2014). Plasmonic properties of silver nanoparticles embedded in diamond like carbon films: influence of structure and composition. Appl Surf Sci.

[15] Mishra YK, Mohapatra S, Singhal R, Avasthi DK, Agarwal DC, Ogale SB (2008). Au–ZnO. A tunable localized surface plasmonic nanocomposite. Appl Phys Lett.

[16] Manish K, Tanuj K, Avasthi DK (2015). Study of thermal annealing induced plasmonic bleaching in Ag:TiO2 nanocomposite thin films. Scr Mater.

[17] Mohapatra S, Mishra YK, Ghatak J, Kabiraj D, Avasthi DK (2008). Surface plasmon resonance of Ag nanoparticles embedded in partially oxidized amorphous Si matrix. J Nanosci Nanotechnol.

[18] Manish K, Suchand Sandeep CS, Kumar G, Mishra YK, Philip R, Reddy GB (2014). Plasmonic and nonlinear optical absorption properties of Ag:ZrO2 nanocomposite thin films. Plasmonics.

[19] Borges J, Buljan M, Sancho-Parramon J, Bogdanovic-Radovic I, Siketic Z, Scherer T, Kübel C, Bernstorff S, Cavaleiro A (2014). Evolution of the surface plasmon resonance of Au:TiO2 nanocomposite thin films with annealing temperature. J Nanoparticle Res.

[20] Bazioti C, Dimitrakopulos GP, Kehagias T, Komninou P, Siozios A, Lidorikis E, Koutsogeorgis DC, Patsalas P (2014). Influence of laser annealing on the structural properties of sputtered AlN:Ag plasmonic nanocomposites. J Mater Sci.

[21] Yaremchuk I, Meškinis Š, Fitio V, Bobitski Y, Šlapikas K, Čiegis A, Balevičius Z, Selskis A (2015). Spectroellipsometric characterization and modeling of plasmonic diamond-like carbon nanocomposite films with embedded Ag nanoparticles. Nanoscale Res Lett.

[22] Yaremchuk I, Tamulevičienė A, Tamulevičius T, Šlapikas K, Balevičius Z, Tamulevičius S (2014). Modeling of the plasmonic properties of DLC-Ag nanocomposite films. Phys Status Solidi (a).

[23] Louroa C, Wagner MC, Carvalho N, Stueber M, Cavaleiro A (2011). Thermal stability in oxidative and protective environments of a-C:H cap layer on a functional gradient coating. Diam Relat Mater.

[24] Rooney P, Rezaee A, Xu S, Manifar T, Hassanzadeh A, Podoprygorina G, Böhmer V, Rangan C, Mittler S (2008). Control of surface plasmon resonances in dielectrically coated proximate gold nanoparticles immobilized on a substrate. Phys Rev B.

[25] Manninen NK, Ribeiro F, Escudeiro A, Polcar T, Carvalho S, Cavaleiro A (2013). Influence of Ag content on mechanical and tribological behavior of DLC coatings. Surf Coatings Technol.

[26] Maier SA (2007). Plasmonics: fundamentals and applications. Springer Science & Business Media

[27] Khlebtsov NG (2008). Optics and biophotonics of nanoparticles with a plasmon resonance. Kvant Electron.

[28] Vasilevskiy M (2000). Effective dielectric response of composites containing uniaxial inclusions. Phys Status Solidi B.

[29] Malitson IH (1965). Interspecimen Comparison of the Refractive Index of Fused Silica. JOSA 55(10):1205-1209. http://www.slac.stanford.edu/grp/eb/dircrd/melles-griot-fused-silica.pdf

[30] Johnson P, Christy R (1972). Optical constants of the noble metals. Phys Rev B.

[31] Wang HH, Liu CY, Wu SB, Liu NW, Peng CY, Chan TH, Hsu CF, Wang JK, Wang YL (2006). Highly Raman-enhancing substrates based on silver nanoparticle arrays with tunable sub-10 nm gaps. Adv Mater.

[32] Yizhuo C, Banaee MG, Crozier KB (2010). Double-resonance plasmon substrates for surface-enhanced Raman scattering with enhancement at excitation and Stokes frequencies. ACS Nano.

[33] Lin VK, Teo SL, Marty R, Arbouet A, Girard C, Alarcon-Llado E, Liu SH, Han MY, Tripathy S, Mlayah A (2010). Dual wavelength sensing based on interacting gold nanodisk trimers. Nanotechnology.

[34] Kvasnička P, Homola J (2008). Optical sensors based on spectroscopy of localized surface plasmons on metallic nanoparticles: sensitivity considerations. Biointerphases.

